# Incentivizing Immunity: Black and Latinx Individuals’ Attitudes About COVID-19 Vaccine Incentives

**DOI:** 10.1007/s40615-025-02550-2

**Published:** 2025-07-22

**Authors:** Fernanda L. Cross, Charles E. Williams, Ana Patricia Esqueda, Rebecca Hunt, C. P. Ku, Joel Lucio, Sarah Bailey, Susie Williamson, Erica E. Marsh, Kenneth Resnicow

**Affiliations:** 1 School of Social Work, University of Michigan, Ann Arbor, USA; 2 Department of Psychology, University of Michigan, Ann Arbor, USA; 3 Michigan Institute for Clinical & Health Research, University of Michigan, Ann Arbor, USA; 4 Department of Obstetrics & Gynecology, University of Michigan, Ann Arbor, USA; 5 Bridges into the Future, Ann Arbor, USA; 6 Core Well Health, Ann Arbor, USA; 7 Department of Obstetrics and Gynecology, University of Michigan Medical School, Ann Arbor, USA; 8 Health, Department of Health Behavior and Health Education, University of Michigan School of Public Health, Ann Arbor, USA

**Keywords:** COVID-19 vaccine, Black, Latino, Community-based participatory research, Vaccine incentive

## Abstract

**Learning Objective:**

Identify drivers of COVID-19 initial and booster vaccinations.

**Background:**

Minority communities were disproportionately impacted by COVID-19, yet many expressed vaccine hesitancy. While a combination of incentivized strategies, namely financial and workplace rewards or mandates, seems to have boosted the rate of vaccination, little is known about the role of vaccine incentives in Black and Latinx communities specifically. Drawing from postcolonial theory and health belief model, our goal was to identify, through qualitative interviews, the perceptions of incentives, such as financial, travel, and employment related, in encouraging vaccination amongst Black and Latinx communities.

**Methods:**

Interviews were conducted between 2022 and 2023 with 24 Black and 10 Latinx Michigan residents self-reported as up-to-date (2022, completed primary series; 2023, completed bivalent booster) and not up-to-date (2022, incomplete primary series; 2023, no bivalent booster). We used a community-based participatory research approach, collaborating with 15 community-based organizations to develop research questions, interview protocols, and methods for data collection, and analysis. Thematic coding of interviews was conducted.

**Results:**

Participants reported that work and travel-related incentives, including bonuses, maintaining employment, and being able to travel, were successful in supporting their decision to vaccinate. Financial incentives were mainly viewed negatively and as causes for suspicion and subsequently disincentivized some participants to take the vaccine.

**Conclusion:**

Our results indicate that financial incentives were a less effective way to drive uptake among the Black and Latinx Michiganders we interviewed. Understanding the potentially negative effects of vaccine financial incentives is essential to combat vaccine hesitancy. It is critical to ensure vaccine incentives align with community values and preferences and do not further damage trust in public health.

The disproportionate impact of the COVID-19 pandemic on Black and Latinx communities has been widely documented [1]. From the greater number of cases, hospitalizations, and mortality rates, to impacts across employment, mental health, and social relationships, these communities were at greater risk throughout the pandemic [2–4]. However, while effective vaccines have been produced uptake is lower in these communities suggesting vaccine hesitancy [5–7]. To increase vaccination rates several forms of incentives, ranging from financial rewards to work and travel mandates, were introduced [8]. However, little is known about Black and Latinx individuals’ perceptions of incentives encouraging COVID-19 vaccination, and how such perceptions may relate to historical oppression and medical mistrust.

## Theoretical Framework

We draw on postcolonial theory and the health belief model to help us understand the role of incentives in vaccination among Black and Latinx individuals. Utilizing postcolonial theory, a cross-discipline theory that intersects humanities, culture, power, inequality, and knowledge, we identified elements to help interpret results from respondents and understand the actions of policymakers [9, 10]. The literature for postcolonial theory provides a critical framework for understanding vaccine hesitancy among Black and Latinx communities, especially in response to mandates and incentives. Historical experiences of medical exploitation and coercion from unethical experimentation to structural neglect, continue to shape how health interventions are received [11–13]. What may appear as neutral public health strategies, such as vaccine mandates or financial rewards, are often interpreted as disciplinary tools tied to access to work, school, or mobility [14].

These strategies may lead to compliance without trust, as individuals make decisions under structural pressure. Additionally, public health messaging that lacks cultural grounding often contributes to othering, reinforcing mistrust and distancing communities from health systems [15]. Further, critiques of universalism in behavioral economics suggest that financial incentives may be ineffective or counterproductive in racialized communities, where such approaches fail to account for cultural context and historical trauma. Postcolonial theory helps us understand the Western eurocentric priorities that arise in the development and planning of health education programs, the lack of access to healthcare or the political and economic systems that plan and administer health services, as well as the healthcare disparities experienced by marginalized communities [16, 17].

In contrast, when analyzing those who reported taking action, we used the health belief model (HBM), a foundational theory used to explain health-related behaviors, particularly in preventive health contexts such as vaccination. The model posits that individuals are more likely to take health action such as receiving a vaccine when they perceive a health threat as serious (perceived severity), believe they are personally vulnerable to it (perceived susceptibility), recognize the benefits of taking action, and view the barriers to that action as low [18]. HBM also considers the role of cues to action, such as mandates or provider recommendations, as well as self-efficacy, or confidence in one’s ability to act. When perceived barriers outweigh benefits due to fear, mistrust, or logistical challenges individuals are less likely to take preventive action, even if they acknowledge the threat itself. In the context of COVID-19 vaccination, HBM has been applied to understand how concerns about safety, side effects, and institutional distrust influence vaccine hesitancy, especially in marginalized populations.

In focusing on the effectiveness of incentives, postcolonial theory helps explain why hesitancy persists among Black and Latinx communities by foregrounding the impact of structural inequalities, historical injustices, and systemic exclusion. In contrast, the HBM helps explain the conditions under which individuals choose to act despite these barriers.

## Incentive Campaigns

Since the release of the COVID-19 vaccines in the USA in 2021, the effectiveness of incentive campaigns for vaccine updates has been a major concern for public health [8, 19–21]. Typically offered to communities by institutions, such as governments, schools, and workplaces, these campaigns included financial, travel, work or school-related, and opportunity-based incentives to contribute to community vaccination levels [8, 19–22]. The state of Michigan, for example, offered $50 to residents who helped others attend their vaccine appointments [8]. Additional vaccine campaigns offered unique opportunities, such as the chance to win free tickets (e.g., concerts, amusement parks, and sports events), the ability to drive on race car tracks, dinner with public officials, and free access to state parks or free drinks and meals from restaurants upon providing proof of vaccination [8].

In an analysis by Carpio and colleagues, higher monetary incentive amounts were associated with higher willingness to get vaccinated, with incentives in the $600–700 range yielding the highest vaccine efficacy levels [23]. On the other hand, lottery-based incentive programs were tied to more mixed effectiveness. For example, the State of Ohio held its Vax-a-Million lottery in 2021, which gave those who received the vaccine a chance to win a large monetary prize [19]. In subsequent analyses of the lottery, Mallow and colleagues found that there was a small increase in vaccinations that could be attributed to the lottery, while other studies found no statistically significant change [20, 21]. Some have speculated that vaccine incentives may reinforce negative ideas about vaccines and convince individuals to forego the vaccine as they may cancel out autonomous motivation due to being perceived as controlling or coercive [22, 24, 25]. However, a systematic review by Khazanov and colleagues found that vaccine incentives did not negatively impact vaccine uptake [26].

Another type of incentive program to increase vaccine uptake was requiring updated vaccination to travel or attend in-person venues and events, requirements which seemed to be perceived positively according to a nationally representative sample of adults US including Black and Hispanic demographics [27]. Likewise, workplace incentive campaigns were yet another way that institutions sought to increase employee vaccination rates. A study by Berry and colleagues compared staff vaccination rates between different nursing home facilities and found that employers’ incentive campaigns were correlated with increased vaccine uptake [28]. Rather than recommending a specific strategy, the study found that the facilities that rewarded staff with prizes such as t-shirts and other merchandise had higher rates of coverage [28].

While monetary incentives for the COVID-19 vaccination were seen by many individuals as a novelty, vaccine incentives have existed for decades [29]. A meta-analysis of interventions to improve influenza and pneumococcal vaccination rates among adults showed that incentives that eliminated out-of-pocket costs in a pay environment appeared to be more effective than those providing a small reward [30]. On the other hand, in a randomized controlled trial of injection drug users, monetary incentives were superior to increased outreach in achieving adherence to a multi-dose hepatitis B vaccine series [31]. Similarly, in a randomized controlled trial of HPV vaccinations for 16- to 18-year-old girls, letters including the offer of vouchers worth $73 for undergoing 3 vaccinations were significantly more effective than standard invitation letters in increasing vaccination completion [32]. Finally, in a randomized cluster trial aimed at minimizing absenteeism resulting from influenza among 12,222 employees, nominal incentives of $5 improved vaccination rates in the workplace [33]. Previous qualitative research suggests that these communities have good reason to feel hesitant to get vaccines [5, 34–38]. Among the chief reasons participants in other qualitative research have cited includes a history of mistreatment in the medical system, conspiracy theories which ran rampant throughout the pandemic, and poor communication of public health information throughout the pandemic [5, 11, 38–43]. Notably, rates among Latinx communities appear to have increased, closing the gap compared to Whites, yet vaccination uptake for COVID-19 boosters continue to lag behind, suggesting ongoing barriers in receiving vaccinations [44, 45]. In spite of this wealth of literature on the subject of vaccine hesitancy, the degree to which financial incentives are effective in Black and Hispanic communities is less well-researched. This study seeks to understand whether financial incentives are effective, and to what degree, in increasing COVID-19 vaccination rates among Black and Hispanic populations.

## Childhood Vaccination Requirements

From a larger vaccine perspective, during the first 24 months of life, the Advisory Committee on Immunization Practices (ACIP) recommends routine vaccinations against 14 diseases, including poliovirus, hepatitis A and B, measles, varicella, mumps, and rubella, and flu vaccinations during every flu season [46]. Many of these recommendations become requirements, though varied by state, as schools require vaccines for entry [47]. All 50 states require the DTap vaccine as well as vaccines against polio, varicella, measles, and rubella; 49 states require vaccination against mumps; 44 states require the hepatitis B vaccine; and 17 states require the hepatitis A vaccine [47]. Among children born during 2018–2019, vaccination coverage by 24 months was 90% for all recommended vaccines except hepatitis A and the influenza vaccine, which came in at 70% [48]. Nonetheless, coverage among those underinsured or insured by Medicaid was lower than that among privately insured children [48]. Likewise, compared to White children, overall vaccine coverage was lower among Black and Latinx children [48]. Interestingly, a systematic review of financial incentives on coverage and uptake of health interventions targeting children under 5 years of age showed that the most pronounced effects are achieved by programs that remove user fees for access to health services, followed by those that condition financial incentives on participation in health education and attendance to healthcare visits, demonstrating the positive impact of financial incentives associated with health programs [49].

## Financial Incentives for COVID-19 Within Black and Latinx communities

Most recently, COVID-19 vaccination rates among Black and Latinx communities have been consistently lower compared to non-Hispanic White communities [50]. Factors contributing to general vaccination hesitancy include knowledge gaps, geographic location, structural inequalities, such as healthcare access and language barriers, as well as misinformation, government mistrust, and concerns about vaccine efficacy and their side effects [5, 7, 41, 51]. As a result, there have been efforts to increase vaccination rates among Black and Latinx groups. This includes collaborations with community leaders, increasing proximity to vaccination sites, and providing financial and non-financial incentives [52, 53]. However, there appear to be mixed results regarding the efficacy of financial incentives, especially when considering the historically marginalized experiences of Black and Latinx communities. Conflicting results may be due to differences in the sample’s political affiliation, age, and confidence in vaccines [54, 55].

For example, a study with low-income participants enrolled in Medicaid (*n* = 2701), 30% and 33% of which were Black and Latinx, respectively, found that financial incentives of either $10 or $50 were insignificant to increase vaccination rates [55]. However, they found that financial incentives reduced vaccination rates, albeit only among men over 40 and those who expressed support for Trump in the 2020 presidential election [55]. Moreover, a pilot study exploring the efficacy of financial incentives among Black and Latinx populations, specifically, found that a $10 or $50 incentive provided no significant vaccine uptake after 30 days; however, using predictive models and out-of-survey data, they hypothesized that larger incentives of about $140–$209 may increase vaccination uptake among Black and Latinx communities [56]. This suggests that higher amounts may motivate Black and Latinx groups to increase vaccinations.

Furthermore, a study with a nationally representative sample found that lottery-based incentives with prizes of at least $1 million also increased vaccination rates whereas smaller financial incentives appeared to be ineffective [57]. They reported lottery programs had varied success in vaccination uptake among states, with the unsuccessful states being heavily Republican, highlighting other sociopolitical factors possibly mediating vaccination uptake [26, 57, 58]. This is in line with other work that has indicated certain demographics, such as self-identifying as Republican or white, as well as being of older age, are more likely to be resistant to being vaccinated [55, 59].

On the other hand, financial incentives appeared to be more effective in low-income and younger groups, as well as those with greater confidence in vaccines overall [26]. Experts have previously expressed concern that people with low income and limited choices may perceive financial incentives as coercive [60]. However, recent studies have demonstrated that moderate financial incentives ($140–209, $1000) appear to increase vaccination uptake among Black and Latinx communities [56, 61]. Meanwhile, larger incentives of about $1500 appeared counterproductive in Black and Latinx groups, further demonstrating how financial incentives may be effective, though within a certain range of compensation [62].

Much of the current literature focuses on samples representing the general population in the USA. Given that there have been and continue to be COVID-19-related health disparities for Black and Latinx populations in the USA, a deeper focus on perceptions of vaccine incentive campaigns among these groups is overdue [1, 63]. In this study, Black and Latinx individuals of varying vaccination statuses were interviewed to better understand their perspectives on how different vaccine incentives impacted their decisions about the COVID-19 vaccine.

## Methods

We conducted 34 virtual interviews with Black and Latinx residents of Michigan. Following a community-based participatory research (CBPR) model, our study team formed a partnership between researchers and a Steering Committee (SC) comprising 16 community leaders from various organizations. The SC played a pivotal role in shaping research questions, interview protocols, procedures, participant recruitment, and data interpretation. Participants were recruited with the support of SC members who distributed digital flyers within their networks. Interested individuals contacted the study team via phone or email. Participants answered a few brief eligibility questions asking about their race, ethnicity, and COVID-19 vaccination status.

Each interview lasted approximately 30–40 min, and participants were compensated with a $50 gift card (increased to $75 for interviews conducted in 2023). To be eligible, individuals had to (1) be 18 years or older, (2) self-identify as Hispanic/Latinx or Black/African American, and (3) be up-to-date in the vaccines (received the first two doses of the vaccine released in 2021) or not up-to-date (received only one dose of the vaccine or were unvaccinated). Given changes in the CDC guidelines to reflect the new COVID-19 booster introduced in the Fall of 2022, our eligibility criteria was updated accordingly.

For interviews conducted before January 1 st 2023, participants were considered *up-to-date* if they had completed the primary series released in 2021 and *not up-to-date* if they had not received the primary series. Participants interviewed after January 1 st 2023 were considered *up-to-date* if they had completed the primary series from 2021 and the bivalent booster released in October 2022. They were considered *not up-to-date* if they had not received the bivalent booster. A total of 174 individuals were approached, 40 were consented, and 34 interviewed. Twenty-four of our participants identified as Black and ten as Latinx (total *n* = 34). They were between the ages of 21 and 74 (*mean* age = 41). Nineteen participants were considered up-to-date on their vaccines, and 15 not up-to-date following the CDC guidelines. See Table 1 for additional demographic information.

**Table 1:** Participant demographics

	*n*	%
County		
Wayne	8	29.6%
Washtenaw	12	44.4%
Kent	5	17.9%
Other	2	7.2%
Race		
Black/African American	21	77.8%
Mixed race	2	7.4%
Unknown	4	14.8%
Ethnicity		
Hispanic/Latino	6	22.2%
Not Hispanic/Latino	12	44.4%
Unknown	9	33.3%
Age		
18–29	6	22.2%
30–59	15	55.6%
60 +	6	22.2%
Gender		
Female	23	85.2%
Male	4	14.8%
Vaccination status		
Up-to-date	20	74.1%
Not up-to-date	7	25.9%

Verbal consent was obtained during phone calls following eligibility assessment. Interviews were conducted in either English or Spanish based on participant preference (31 in English, 3 in Spanish). Interviews were recorded, transcribed, and professionally translated. Anonymized Spanish transcripts were professionally translated into English. Bilingual research staff reviewed all transcripts for accuracy. We engaged in a phenomenological approach aiming to better understand and describe participant’s experiences with and perspectives about the COVID-19 vaccine [64]. Interview questions inquired about participant’s vaccine related decisions, whether their decisions changed throughout the pandemic, and reasons for getting or not getting vaccinated, which often elicited participant’s description of incentives they considered when making their decision. For instance, one of our questions asked “What are some reasons for getting the vaccine that you have considered?” We also asked about whether such decisions impacted relationships with those around them. We observed a saturation point after approximately 30 interviews, where no new codes, themes, perspectives, or insights emerged, suggesting that our sample size was adequate to comprehensively understand the study’s focus areas. Data analysis involved three researchers engaging in inductive coding to identify interview themes. A thematic codebook was developed based on initial analysis and weekly discussions with the analytical team, undergoing revisions throughout the analysis process to ensure quality and rigor. All analyses were conducted using Dedoose Version 9.0.17. Inter-rater reliability (IRR) for code assignment yielded a score of 0.85, indicating strong reliability. The research procedures were approved by the University of [blind for review]. Institutional Review Board (HUM00198399).

## Results

Participants were asked several questions that assessed their decision-making process regarding the COVID-19 vaccine as well as some of the factors that impacted their decision, including whether being offered a cash prize would make a difference. Our findings yielded three incentive-related themes that highlighted the motivational approaches that factored into participants’ decisions to receive or not receive the COVID-19 vaccine: (1) financial incentives, (2) instrumental support, and (3) mandates and disincentives. Overall, despite good intentions, not all incentives were well-received by participants. Participants who were not-up-to-date on their COVID-19 vaccines were far more likely to express reluctance about financial incentives.

### Theme 1: Financial Incentives

One of the incentive areas participants reported nearly unanimous opposition to was cash prizes for getting vaccinated. In particular, participants reported concerns about the motives behind cash incentives. This, combined with the vaccine’s recent development, left some participants feeling as though they were subjects in an experiment, rather than being encouraged to get vaccinated. Participants repeatedly cited concerns with the fact this was the first time that they had ever heard of cash incentives for getting vaccinated. This raised concerns about the potential risks of receiving the vaccine:

*“I just feel like…that made me more leery about it because they’re paying you to get a vaccine…are they trying to literally poison us? Because who would pay somebody to get a vaccine? They've never paid us to get flu vaccines or tuberculosis and all those…And to pay somebody to get a vaccine, it just was really sketchy.”* Latinx participant, age 35, not up-to-date, 2023

In addition to feeling uneasy about the unprecedented nature of these payments, participants also reported feeling as though they were being experimented on. Participants’ reactions evoke a form of “double consciousness” articulated by W.E.B. Du Bois and later echoed by Frantz Fanon, a critical awareness of being both an object of care and a target of control, shaped by histories of racial subjugation and colonial domination [65, 66]. This led to the conclusion that if the government had this money to help people who need it, it should not have strings attached. Instead, cash incentives are seen as instruments of biopolitical power or mechanisms of control [67, 68]. Further financial incentives activated historical memory, evoking comparisons to experimentation and systemic devaluation of life [11, 12, 40].

*“Pay poor people to experiment on themselves is what I heard. You know that we need this money and so you hold the money hostage. If you can just give me the money, give me the money because I need the money. Don’t tie it to this vaccine that we're not sure what the long-term impacts or effects will be. Heard lots of, “Oh they always want to experiment on poor black people.”* Black participant, age 43, not up-to-date, 2023

Simultaneously, participants acknowledged that a larger amount of money instilled greater doubt about the safety and efficacy of the vaccine, while also not being enough money to be worthwhile. Participants also highlighted concerns about long-term side effects and a payment of $50–$100 not being adequate to compensate people for something that they felt was unsafe. From a postcolonial perspective, these perceptions are shaped by a history in which healthcare interventions have often been deployed not as care but as opportunities for profit, reinforcing exploitative dynamics where health becomes commodified and communities become markets [12, 35, 36].

*“Here’s a hundred dollars to be a test subject in extreme cases…But I’m sure that if they wanted to offer anything more than a hundred dollars, that would be them confirming that they’re not very confident in the decision.”* Black participant, age 38, not up-to-date, 2023

Additionally, participants expressed frustration with the messages sent by cash incentives. This emphasis on financial incentives reflects a postcolonial trait of neoliberal coloniality, where health decisions are framed as economic transactions, prioritizing compliance over care [13, 69]. Responses indicated that this method of encouraging COVID-19 vaccination was disingenuous:

*“They had all types of stuff on the radio, all types of stuff. But they was saying, if you vaccinated, you could win prizes and stuff. And to me, it was deterring from the fact that we need to get... we shouldn’t have to pay you all to get healthy, to do what you all need to do …To me, it’s sending the wrong message. For one, it’s already free. You don’t have to pay for it. So that should be it right there. I shouldn’t have to do…anything. You shouldn’t have to win a prize or anything for it.”* Black participant, age 29, up-to-date, 2022

Perceptions of cash incentives for vaccination were primarily, but not exclusively, concerning for the respondents; one participant reported rescheduling their vaccination to a location where cash incentives were paid to those vaccinated. They did not report the cash prize directly influencing their decision to get vaccinated, but rather their choice of location:

*“They were going to schedule me and [vaccine site]. had just started giving vaccines. And then, one of my friends said that they was paying you $50 in [city]. to get the vaccine, which was the same day we were scheduling for me to go to [city]. I canceled [city]. and he took me... I’m in [city]. so we went down to the COVID center.”* Black participant, age 57, up-to-date, 2022

### Theme 2: Instrumental Support

Broadly, participants were willing to get vaccinated if it meant a return to more normal activities, including international travel. This was true even for participants who felt as though the vaccine would have a negative impact on their health. Being able to travel and attend events was a strong enough incentive for participants to consider putting some feelings of hesitancy aside. This pattern aligns with the health belief model, which suggests that individuals weigh perceived benefits against perceived barriers when making health decisions [18]. In this case, the perceived benefit of regaining access to valued activities outweighed concerns about side effects or distrust. Additionally, travel and event participation requirements served as powerful cues to action, external triggers that prompted behavior change even among those who were previously hesitant.

*“If I travel maybe, that’s what kind of precipitated my initial booster was I was going to a conference and the conference required it, and that kind of outweighed my own hesitancy and I got it... I think that’s probably the only case that I would, if something that I was doing mandated it. If I was traveling internationally and they required it.”* Black participant, age 43, not up-to-date, 2023

In addition to reporting more willingness to get vaccinated in light of a requirement for travel, participants were also more accepting of a vaccine if the alternative was extremely inconvenient. For example, one participant who had a cruise planned viewed getting a single vaccine dose as far less of a hassle than paying for and repeatedly taking COVID tests throughout the trip. This decision reflects core constructs of the Health Belief Model, particularly perceived barriers and perceived benefits [18]. The inconvenience and financial cost of repeated testing were seen as greater barriers than the discomfort or risk associated with vaccination. In turn, the vaccine became a more appealing option, not necessarily because participants believed strongly in its health benefits, but because it represented the most efficient path to achieving their goal:

*“When we went on a cruise last year we had to show proof of vaccination, or I believe we would have to go through... Either they weren’t going to let us on, or we would have to take numerous COVID tests that were, I believe some of the more expensive kinds too.”* Latinx participant, age 50, up-to-date, 2023

Participants who reported receiving the initial series of vaccines due to travel requirements reported that since these requirements are no longer being enforced they feel far less inclined to stay up-to-date:

*“We also got them because they were a requirement for travel mainly. So now that you can travel anywhere without it, why would you still get them?” Latinx participant, age 47, not up-to-date, 2023*

### Theme 3: Mandates and Disincentives

Participants reported that work or school mandates were mostly successful for their vaccination. Though from a postcolonial perspective, participants’ experiences with COVID-19 vaccine mandates and disincentives reflect ongoing legacies of structural coercion, medical paternalism, and the commodification of health within marginalized communities. Many participants described getting vaccinated not out of trust or conviction, but because compliance was tied to economic survival, recurring theme in postcolonial critiques of public health systems that operate more as instruments of control than care [13, 70, 71]. One college student reported that a school requirement would have made them decide to get vaccinated:

*“I’m a college student, so at one point in time there was a mandate to get vaccinated if you wanted to be on campus. Thankfully at that time I had all fully online classes, so I didn't have to, but if it came down to it to where I needed to get vaccinated to go to class, I definitely would've got vaccinated to go to class. Or when it came to my job, I am not one of those ones to lose my job because I’m trying to make a point. If I needed to get vaccinated, even if it was just one dose, I definitely would've did it.”* Black participant, age 27, not up-to-date, 2023

This idea of putting aside concerns due to the need to keep a job surfaced frequently when participants discussed their reasons for scheduling a vaccine. This shows how the colonial logic of discipline is repackaged in contemporary forms. Vaccine mandates attached to job security, school access, and housing mirror the colonial-era practices in which colonized subjects were required to conform to the health norms and policies of ruling powers to participate in civil society [72, 73]. For many, a requirement for work was a major contributor to their decision:

*“And then I had to [get vaccinated]. just in order to keep my job too, so I had to take that in consideration.”* Black participant, age 30, up-to-date, 2023

Participants whose friends worked in healthcare and participants who were currently working, shared that they faced the risk of termination if they did not get vaccinated. Rather than building trust, these mandates increased overall hesitancy by reinforcing the feeling that their concerns—particularly around efficacy and safety—were being dismissed. The experience contributed to a broader sense of marginalization, where individuals felt they were being spoken for and acted upon, rather than engaged as equal partners in the decision-making process. For this participant, mandating the vaccine felt inappropriate and further deepened their skepticism:

*“A lot of my friends are healthcare workers and they were getting fired if they didn’t get the vaccine. But we didn’t know anything about this vaccine that all of a sudden that they had. And then a lot of people were still getting COVID after they had the vaccine, so what were they really giving them?... And then it was all of a sudden, oh, well, you got to get two rounds of the vaccine, you got to get a booster and you got to get another booster… Just didn’t make sense to me. So me and my children all are unvaccinated.”* Latinx participant, age 35, not up-to-date, 2023

Even in situations where their jobs did not explicitly require vaccination, some participants reported feeling pressure from their workplace to get a COVID vaccine. They recognized that their job duties may have changed without proof of immunization, and were not willing to take a potential pay cut.

*“With my position through my job, that was kind of like, you will get this or you cannot work, or you will work in a different capacity… [not receiving it]. would definitely have affected my paycheck.”* Latinx participant, age 50, up-to-date, 2023

Similarly, in other situations where participants were not explicitly required by their employers to be vaccinated, their jobs played a role in their decision:

*“The majority of us work – at least my friends, we work cleaning houses. So, they also had to get vaccinated to be able to work in the homes where they were. In some houses, we work with elderly people. So, the elderly also ask [if we are vaccinated]. – and they’re right, because of their condition. They don’t have the same strength… and the first thing they would ask you is, “Are you vaccinated already?” Because if you aren’t vaccinated, you can’t go.”* Latinx participant, age 40, up-to-date, 2022

This dynamic reflects what postcolonial scholars such as Gayatri Chakravorty Spivak describe as the silencing of the “subaltern”—where marginalized groups are denied a voice in shaping interventions that directly affect them.^1^ In such contexts, mandates may be experienced not as care, but as coercion [74].

One of the main concerns participants reported was that their providers did not have the same attitudes toward COVID-19 vaccines as with other vaccines. The pressure they felt led them to question the vaccine’s efficacy and safety:

*“For any other vaccine, they didn’t do all of that. They didn’t try to convince you. When I went to my doctor[‘s]. office and they asked me if I wanted to take the flu shot, it was optional. But I feel like with the COVID shot, if I say, ‘No, I don’t want the COVID shot.’ My doctor would literally sit there for 30 minutes and explain everything for me to take this. So it's just for me, I was like, ‘Why is this so serious? Why are they trying to make sure everybody get the COVID shot?’ Cause I feel like I was under a lot of pressure every time I go to my doctor office.”* Black participant, age 27, not up-to-date, 2023

This aligns with postcolonial scholars’ critiques of top-down, one-size-fits-all health campaigns that fail to engage with communities'lived experiences or historical memory. Other participants felt that the public service announcements, requirements, and other incentives or campaigns to increase vaccination coverage made them feel more secure in their conviction not to get vaccinated.

*“I think [matters of vaccination]. should be left a little bit more alone. Stop all the advertisement and the pressure…and maybe more people will get it if they stopped pushing it so hard at the beginning. Every time you go to a doctor’s office, they offer it and offer it. And some work[places]. [it is]. still mandatory. The universities, some of them are still required for dorm living. So I would say stop the mandate or stop the obligation and maybe more people will say, “Okay, fine, I’ll get it every year maybe.”* Latinx participant, age 47, not up-to-date, 2023

These accounts also expose the coloniality of public health messaging, which some participants felt was overbearing and counterproductive. The constant stream of mandates, advertisements, and provider pressure led some to feel more entrenched in their hesitancy.

In spite of reinforcing hesitancy, other participants reported that their providers’ persistence in recommending the vaccine paid off. One participant reported feeling unsure about the decision, but that a second call from the office to schedule the appointment prompted them to decide to get vaccinated.

*“When I got off the phone with my doctor, I still hadn’t made my mind up when I was going to pick up the phone and make the appointment. He had the office people call me back in about 10 minutes to schedule the appointment for me.”* Black participant, age 57, up-to-date, 2022

This excerpt highlights that trust-building and relational engagement are more effective than coercion, and reinforces the postcolonial call for decolonizing health interventions by centering autonomy, relationship, and cultural respect [15].

## Discussion

Our findings demonstrate individuals’ perceptions of COVID-19 vaccine incentives and motivational policies and how they impacted their decision-making, corroborating existing literature, emphasizing the importance of a combination of incentivization and disincentive strategies to increase vaccine acceptance. Financial incentives, vaccine requirements, and workplace rewards for vaccination have been identified as effective incentives [30–33]. However, our study adds depth to this understanding by examining the perspectives of Black and Latinx individuals, a demographic group often overlooked in previous research.

Some studies have shown financial incentives to be moderately effective, while others indicate their impact as small or nonexistent [55–58]. Our findings align with this mixed data, revealing Black and Latinx participants’ skepticism and concerns regarding cash prizes for vaccination. Participants expressed apprehension about the motives behind such incentives, with many feeling uncomfortable about being subjects in an experiment or coerced into vaccination, especially when provided lower compensation ($50–$100) for higher perceived risks. This aligns with previous research that found $50–$100 financial compensation was ineffective at increasing vaccination rates among Black and Latinx Medicaid holders [55]. This skepticism was particularly pronounced among Black participants in our study, reflecting a deep-seated mistrust rooted in historical experiences of medical racism, supporting previous research [5, 39, 41, 75, 76]. Additionally, our study highlights the influence of various contextual factors on individuals'decisions regarding vaccination. For instance, instrumental support such as the expectation of vaccination for travel was generally well-received by participants, indicating a willingness to prioritize the return to normalcy over concerns about vaccine safety [27].

Similarly, workplace requirements for vaccination were effective in prompting individuals to get vaccinated, underscoring the role of external mandates and disincentives in shaping behavior. However, work or school mandates also generated mixed feelings, with some participants feeling pressured to prioritize their jobs or school over their personal beliefs. This indicates that work or school mandates are not always enough to increase uptake, consistent with the findings of Berry and colleagues’ report that employees are more responsive to campaigns in which additional incentives are offered [28]. Interestingly, the influence of healthcare providers on vaccination decisions emerged as a significant theme in our study. While some participants reported feeling pressured or coerced by their doctors to get vaccinated, others appreciated the guidance and encouragement provided by healthcare professionals. Our findings partially support past studies that found that Black and Latinx individuals were likely to trust their doctors’ recommendation to get vaccinated [37]. The divergent experiences reported by participants underscore the importance of nuanced communication and patient-centered care in addressing vaccine hesitancy.

To fully interpret these patterns, we applied two theoretical frameworks: postcolonial theory and the HBM [10, 17, 77, 78]. A postcolonial lens allows us to see how skepticism toward vaccine incentives among Black and Latinx participants is shaped by a long history of medical exploitation, systemic racism, and being “acted upon” rather than engaged. Vaccine mandates tied to job security, school access, or housing mirror colonial-era practices where compliance with health norms was a condition for inclusion in society. Participants’ experiences echoed the colonial logic of discipline, repackaged in modern institutions that continue to marginalize racialized communities. Such strategies, while framed as public health, may inadvertently replicate patterns of coercion and exclusion, particularly when accompanied by a lack of relational trust or community engagement [12, 70, 71, 73, 74].

As it relates to the HBM most directly aligned with participants’ responses to instrumental support, suggest tangible, situational motivators such as travel requirements, employment expectations, and the avoidance of logistical burdens like testing. Participants were more likely to get vaccinated when it enabled them to choose access valued experiences. In these cases, the perceived benefit of travel and cues to action**,** such as vacation and conference policies, prompted vaccine uptake, even among those who expressed safety concerns or vaccine hesitancy [18]. Importantly, while these decisions were shaped by policies, they were often perceived by participants as autonomous and rational choices made in response to practical life circumstances. Unlike responses to financial incentives or strict mandates, which were often perceived as coercive or manipulative, instrumental support provided a sense of control, allowing participants to weigh risks and benefits in a way that preserved their agency, even in constrained conditions.

### Implications for Public Health and Future Directions

Our findings suggest the urgent need for tailored interventions integrating culture-affirming strategies to decrease hesitancy and increase COVID-19 vaccination uptake among Black and Latinx populations [79]. This study highlights that the promotion of mandates and the offering of financial incentives, while intended to increase vaccine uptake, can paradoxically contribute to increased hesitancy, particularly among Black and Latinx individuals. When such strategies are perceived as coercive, transactional, or dismissive of community experiences and concerns, they may reinforce distrust rather than build confidence. When relying solely on financial incentives or punitive mandates, interventions should take into account the complex interplay of social, cultural, and historical factors that shape individuals’ attitudes and behaviors toward vaccination and other health behaviors. Collaborative efforts involving community leaders, healthcare providers, and public health officials are essential for building trust, addressing concerns, and promoting vaccine acceptance within these communities [80]. For example, during the heat of the pandemic, the Community Outreach Pilot Project was able to reach Latinx Michiganders by collaborating with religious organizations, academics, CBOs, and community members [81]. Partners with pre-established trust in their communities were able to come together, pool their resources, and reach more than two thousand individuals over the course of the project [81].

Understanding the successes and failures of public health responses to the COVID-19 pandemic is crucial to effectively respond to future public health emergencies. The experiences that people had during the heat of the pandemic have shaped their perspectives long term, for better or worse. For example, work, school, and travel requirements may have been reasons our participants reported choosing to get vaccinated, but participants also reported feelings of fear and doubt surrounding the vaccine that these incentives did not assuage. They felt coerced, and like they were not able to weigh the positives and negatives for themselves, rather than feeling able to trust in the public health system. These efforts to improve trust must be ongoing, not just during times of crisis, and must provide clear, concise, and culturally tailored information [42, 82].

These requirements, in our population, worked insofar as getting vaccines to people, but not in acknowledging the wider systems that have caused distrust of public health. It is not enough to just get vaccines into arms. We, as public health practitioners, must work to ensure that health information we disseminate is trusted and effective, so that future crises are not so polarizing.

### Limitations

This qualitative study is not generalizable to the entire population of Michigan, nor is it representative of all Black and Latinx voices within the state, which limits the generalizability of findings to others residing in varied parts of the country. Additionally, the sample is predominantly female (85.2%). As such, the viewpoints captured in our interviews largely represent the feelings of female community members thus presenting a more limited perspective on individual’s COVID-19 vaccine related decisions. Additionally, young adults’ feelings toward incentives may not be fully captured in these interviews for the same reason, with 77.8% of the sample being 30 or older. Lastly, we changed our recruitment language from recruiting “vaccinated and unvaccinated” individuals to recruiting individuals who were “up-to-date” or “not up to date” on their vaccination status, following the CDC’s changing definition. While this change in recruitment language did not impact our results, it prevented our team from having 100% certainty prior to the interview on whether or not participants who were considered “not up to date” had in fact received one dose of the vaccine or none. During the interviews this distinction was made clear since participants always disclosed if they had received the vaccine and how many doses they received. In spite of these limitations, though, the individual perspectives captured through this project are vital to consider. This is particularly important in Michigan, where Black and Latinx residents of the state suffered disproportionately as a result of the pandemic, despite making up a smaller portion of the state’s population [83, 84]. Future research could incorporate both a larger sample size and a mixed-methods approach to improve generalizability and gather more varied perspectives on this issue.

## Conclusion

This study showcases the ways in which vaccine requirements and incentives played a key role in decisions to receive a COVID-19 vaccine. Additionally, this study highlights a lack of trust in our sample, even among those who chose to get vaccinated. Our participants’ perspectives suggest that vaccine incentives should be informed by the social and cultural contexts of the time. Future public health responses must work to build, and consider, trust when implementing programs and incentives.
